# Synthesis of a Superhydrophobic Fluorinated Nano-Emulsion and Its Modification on the Wettability of Tight Sandstone

**DOI:** 10.3390/ma15114015

**Published:** 2022-06-06

**Authors:** Qiang Li, Zhenzhong Fan, Qingwang Liu, Wenhai Ma, Junliang Li, Nan Li, Pingang Ma, Hongtao Zhang

**Affiliations:** 1 Key Laboratory of Enhanced Oil Recovery, Northeast Petroleum University, Daqing 163318, China; johnlee2013@126.com; 2 Daqing Oilfield Production Engineering Research Institute, Daqing 163453, China; mawenhai@pechina.com.cn (W.M.); junliangli@petrochina.com.cn (J.L.); linanlinan@petrochina.com.cn (N.L.); mapingang@petrochina.com.cn (P.M.); zhanghongtao996@petrochina.com.cn (H.Z.); 3 Heilongjiang Provincial Key Laboratory of Oil and Gas Reservoir Stimulation, Daqing 163453, China

**Keywords:** water blocking, tight sandstone, superhydrophobic, nano-emulsion, wettability, contact angle, spontaneous imbibition

## Abstract

The water-blocking effect is a serious problem when developing tight sandstone gas reservoirs, which can cause a sharp reduction in gas production. Wettability alteration of near-wellbore sand rock surface from superhydrophilicity to superhydrophobicity is an effective method to decrease capillary pressure. In this study, a superhydrophobic fluorinated nano-emulsion was synthesized via a soap-free emulsion polymerization process using methacryloxyethyl trimethyl ammonium chloride, trifluoctyl methacrylate, and styrene as monomers. The effect of the fluorinated monomer concentration on wettability alteration was evaluated by measuring the contact angle of the formation water droplet on the modified glass slides using nano-emulsions with different fluorinated monomer concentrations. The results showed that the nano-emulsion had a good dispersibility and homogeneous particle size of around 90 nm, and with the increase in fluorinated monomer concentration, the contact angle increased. The contact angle was the largest when the fluorinated monomer mass rate concentration reached 50%. The adsorption of nanoparticles could alter the rock wettability from a super hydrophilic state (θ = 7°) to a superhydrophobic state (θ = 150°). The spontaneous imbibition experiments showed that the formation water adsorption quality of the core decreased by 49.7% after being modified by the nano-emulsion. The nano-emulsion showed a good superhydrophobicity and had the potential to be used to reduce the water-blocking damage in the tight gas reservoirs.

## 1. Introduction

A series of issues are closely related to the wettability of rocks when developing tight sandstone gas reservoirs, such as the reservoir damage caused by the lower flow back rate of drilling and fracturing fluids, as well as the low ultimate gas recovery after formation water is trapped in the reservoir [1,2,3,4]. Because of the hydrophilicity and tightness of the rock, the capillary effect is significant, and formation water, injected water, and condensate water are easily retained in the flow channels, resulting in a decrease in the gas phase effective permeability. The lower the original water saturation and permeability of gas reservoirs, the more severe the water-blocking damage, which can lead to a decrease in permeability of more than 80% [5], eventually leading to a decrease or even shutdown of production. This is called the water-blocking effect [6,7,8].

To solve the water-blocking problem for tight sandstone gas reservoirs, it is necessary to reduce the capillary pressure in rock microchannels. As the capillary force is proportional to the surface tension and the cosine of the contact angle, the water-blocking damage can be relieved by using chemicals to reduce the surface tension [9] and change the rock wettability [10]. At present, various types of surfactants, such as cationic, anionic, zwitterionic, and nonionic surfactants, have been researched and are widely used to relieve water-blocking damage [11,12]. However, the performances of different kinds of surfactants are quite different. Some have the problems of a poor compatibility with formation water, poor temperature resistance, or a short validity period. For example, the cationic surfactants will lose their activity in an alkaline environment. At the same time, because of the negative charge on the rock surface, they are easily adsorbed on the rock surface, which will reduce their effect [13]. When anionic surfactants encounter formation water with a high salinity, especially when the content of calcium and magnesium ions is high, the salting-out effect will occur, which changes the molecular structure and fails to achieve a good application effect [14]. While nonionic surfactants have a high stability and are not affected by acids, bases, and salts, the solubility decreases with the increase in temperature under the influence of ether or ester groups. At a certain temperature, the solution starts to become cloudy [15]. For these reasons, more and more researchers are focusing on the research of chemicals that can be used to remove water blocks by changing the wettability of rocks. Wettability has a great effect on critical saturation and relative permeability, which can be significantly improved by altering the near-wellbore rock wettability from strongly liquid wet to intermediate gas wet [16,17,18]. It is well known that the solid phase wettability is related to its surface free energy and roughness [19,20]. To decrease the surface free energy, fluorides such as perfluorobutyric acid, fluorochlorovaleric acid, sodium fluorinated alkyl sulfonate, and perfluorooxa amido quaternary ammonium salt are commonly used as modifiers because of their good stability and low surface energy [21,22,23], and the higher the number of fluoro-groups, the less the wettability of the water phase [24]. With the emergence and development of nanotechnology, nanoparticles are widely used to increase surface roughness because of the volume and surface effects of the small size of the nanoparticles [25,26,27,28]. In 2015, a nanofluid compound made from silicon-based molecules and fluoro-polymer was first reported, which altered the wettability of carbonate and sandstone rocks to gas wetting with a static contact angle of 139° and 138°, respectively. With the emergence of the concept of superhydrophobicity (contact angles greater than 150°) [29], scholars completed vast research using nanoparticles such as nano-silica and nano-titanium dioxide as the wettability modifiers of the rock surface in order to enhance gas production and recovery for their excellent performance in hydrophobicity [30,31]. Among them, nano-silica has better hydrophobic properties and its contact angle can reach 151° [32]. However, these nanoparticles easily agglomerate. producing a large number of hydroxyl unsaturated residual bonds on the surface of the nanoparticles [33]. To improve the dispersibility of these nanoparticles, organic solutions such as methanol and ethanol are often used as solvents [34]. In micro-scale simulation experiments in the laboratory, organic solvents such as methanol and ultrasonic waves are used to ensure the dispersibility of superhydrophobic nano-silica. However, in the field application process, when the superhydrophobic nano-organic solution is injected into the formation it will be diluted by formation water, and the dispersibility of nano-silica will drop rapidly, which may fail to relieve the water blocking damage and may cause secondary damage through the agglomeration of nanoparticles.

This paper aims to solve the problem of the dispersibility of superhydrophobic nanoparticles in water, so that they can be easily applied in field practice. In this paper, a superhydrophobic fluorinated nano-emulsion was synthesized by soap-free emulsion polymerization, which showed a good dispersibility and performance, and could effectively change the wettability of rock, not only reducing the surface free energy of rock, but also changing the rough structure of the rock surface and micropores to prevent the damage caused by the water-blocking effect. The adsorption stability of the nanoparticles and the effect of the fluorinated monomer concentration on wettability were studied, and a comparison experiment considering the spontaneous imbibition of water in the core samples was also studied.

## 2. Experiments

### 2.1. Experimental Materials

Styrene (St), tridecafluorooctyl methacrylate (G06B), methacryloxyethyl trimethyl ammonium chloride (DMC), diethylene glycol dimethacrylate, and 2,2-azobis(2-methylpropylimid) dihydrochloride (V-50) were the analytical reagents (AR), and glass sheets were provided by Chengdu Kelon Reagent Co., Ltd., Chengdu City, China. Pure water was made in the laboratory. Natural tight sandstone core samples from the Hechuan gas field were cylindrical with a diameter of 2.5 cm and a length of around 10 cm. The average porosity of these core samples was 8.69%, and the average permeability was 0.042 mD.

### 2.2. Experimental Methods

#### 2.2.1. Synthesis of the Water-Soluble Superhydrophobic Fluorinated Nanofluids

**Figure 1:**
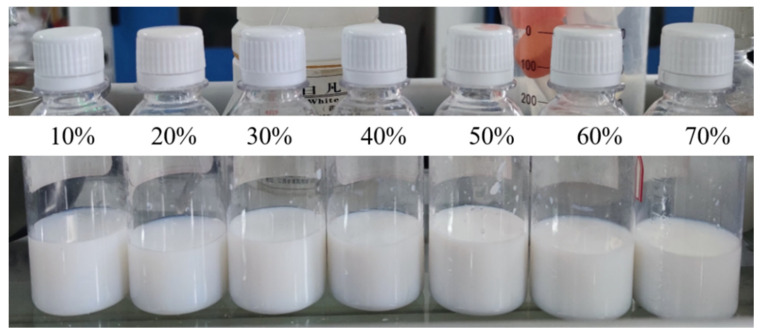
The milky white fluorinated nano-emulsions with fluorinated monomer mass ratios from 10 wt% to 70 wt% after being stored for 30 d.

**Figure 2:**
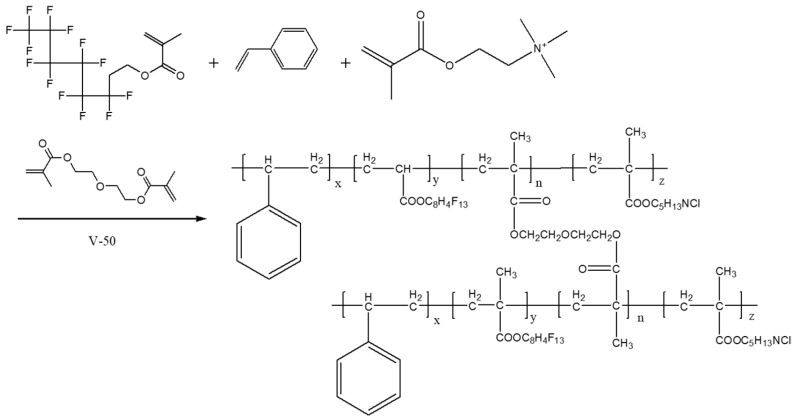
Schematic diagram of the synthesis of the fluorinated nano-emulsion.

To control the emulsion concentration to 10 wt%, the soap-free emulsion polymerization process was adopted for the synthesis of a series of fluorinated nano-emulsions by changing the mass ratio of the fluorine monomer (G06B) from 10 wt% to 70 wt%. Certain mass ratios of pure water, styrene (St), tridecafluorooctyl methacrylate (G06B), methacryloxyethyl trimethyl ammonium chloride (DMC), and diethylene glycol dimethacrylate were filled into a 250 mL three-necked flask. Taking a fluorine monomer concentration of 50 wt% as an example, 4.64 g of styrene, 5 g of tridecafluorooctyl methacrylate, 0.3 g of methacryloxyethyl trimethyl ammonium chloride, and 0.06 g of diethylene glycol dimethacrylate were added into a three-necked flask containing 85 g of pure water, and then the three-necked flask was placed into a 65 °C water bath. The electric stirrer was turned on and set the stirring rate was set at 200–300 r/min. After the temperature of the mixture solution rose to 65 °C, 5 g of 2,2-azobis(2-methylpropylimid) dihydrochloride (V-50) was added to the mixture solution as an initiator. Meanwhile, the temperature of the water bath was adjusted to 80 °C, and the synthesis reaction occurred while being stirred at this constant temperature for 4 h. After cooling down, milky white fluorinated nanofluids were obtained (Figure 1) and there was no stratification, agglomeration, flocculation, and demulsification found in these nano-emulsions after being stored for 30 d. The results showed that the dispersibility of these nano-emulsions was good. The schematic diagram of the synthesis of fluorinated nanoparticles is shown in Figure 2.

#### 2.2.2. Measurement of the Molecular Structure of the Nano-Emulsion

The WQF-520 Fourier Transform Infrared Spectrometer from the Beijing Beifen Rayleigh Instrument Company was used to characterize the molecular structure and chemical composition of the fluorinated nanoparticles. The sample preparation method was as follows: ground potassium bromide was first pressed into tablets, and then the fluorinated nano-emulsion was dropped onto the potassium bromide flakes and dried in the oven to remove the water.

#### 2.2.3. Characterization of the Nanoparticle Size and Morphology

The particle size distribution of the fluorinated nano-emulsion was measured using a BI-200 M wide-angle laser scattering instrument from Brookhaven Instruments, New York, NY, USA, and the morphology of the fluorinated nanoparticles and the core surface before and after treatment was observed using a Quanta 450 scanning electron microscope of FEI Company, Portland, OR, USA.

#### 2.2.4. Contact Angle Measurement

The glass sheets (25.6 mm × 76.8 mm) and the polished core slices (25 mm × 25 mm × 5 mm) were washed with pure water, absolute ethanol, and pure water for ultrasonic cleaning for 30 min each. After cleaning three times, the glass and core slides were dried in an oven at 110 °C for 8 h. Then, the glass and core slides were soaked in the nano-emulsion for 12 h separately and dried at 80 °C. The SDC-200S contact angle measuring instrument from SINDIN company, Dongguan, Guangzhou, China, was used to measure the contact angle of the formation water on the surface of the glass slides and the cores before and after treatment. The measurement conditions were at room temperature, the droplet volume was 6 μL, and the droplet rate was 100 mm/s.

#### 2.2.5. Measurement of Water Spontaneous Imbibition Volume in Core Samples

To qualitatively evaluate the effect of wettability alteration on preventing core water blocking, the spontaneous imbibition experiment was adopted to compare the water imbibition quality before and after wettability alteration. First, the core was soaked in pure water for 24 h, washed with pure water ultrasonically, dried in an oven at 105 °C for 12 h, and weighted using the analytical balance. Then, the core was placed in a sampling bottle containing 20 mL of formation water for the spontaneous imbibition experiment, and the bottle mouth was sealed with the plastic wrap and a bottle cap. The experiment continued by taking out the core every 1 h, recording its weight after wiping off the surface water, and calculating the mass change of core with time in 8 h. After the first stage of the experiment finished, the core was washed and dried following the same procedures, and then placed in the fluorinate nano-emulsion for 12 h and dried for another 12 h until the core quality no longer changed. The spontaneous imbibition experiment was carried out again to record the water absorption quality change with time.

## 3. Results and Discussion

### 3.1. Effect of Fluorinated Monomer Concentration on the Wettability of Glass Sheets

**Figure 3:**
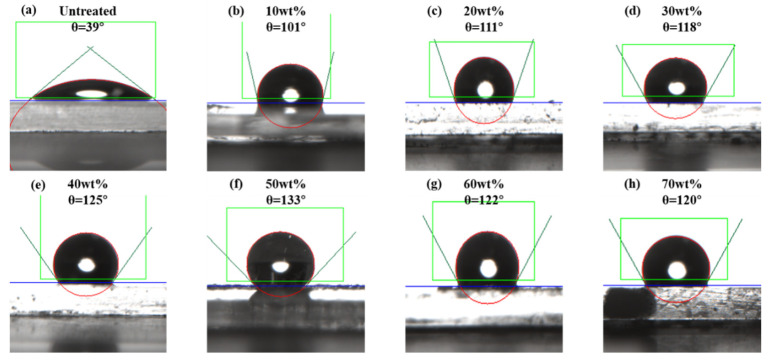
The contact angles on the glass sheets for different fluorinated monomer concentrations: (**a**) untreated glass sheet, (**b**) glass sheet treated with a fluorinated monomer concentration of 10 wt%, (**c**) glass sheet treated with a fluorinated monomer concentration of 20 wt%, (**d**) glass sheet treated with a fluorinated monomer concentration of 30 wt%, (**e**) glass sheet treated with a fluorinated monomer concentration of 40 wt%, (**f**) glass sheet treated with a fluorinated monomer concentration of 50 wt%, (**g**) glass sheet treated with a fluorinated monomer concentration of 60 wt%, and (**h**) glass sheet treated with a fluorinated monomer concentration of 70 wt%.

To study the effect of the fluorinated monomer concentration on the wettability of nanofluids, and to determine the optimal amount of fluorinated monomer, we compared a series of mass ratios for the fluorinated monomer (G06B) from 10 wt% to 70 wt% on the wettability alteration of the glass sheets. The test results are shown in Figure 3.

The contact angle of the formation water on a clean glass sheet was 39°, indicating that the surface of the glass sheet was hydrophilic. After the glass sheets were treated by the nano-emulsions with different concentrations of the fluorinated monomer, the contact angles of the formation water changed significantly.

As the mass ratio of the fluorinated monomer increased, the contact angle of the formation water on the surface of the glass sheets increased and then decreased. When the concentration of the fluorinated monomer was 50 wt%, the contact angle could reach 133°, which was very hydrophobic, and the hydrophobic performance was the best. The formation water droplets on the glass sheet were approximately spherical. This is because the fluorinated nanoparticles could evenly adhere to the surface of the glass slide, providing it with a rough surface structure. At the same time, the nanoparticles were surrounded by fluorinated side chains, which reduced the surface free energy of the glass sheet. With the increase in the concentration of the fluorinated monomer, the number of fluorinated side chains on the surface of the nanoparticles increased and the surface free energy was reduced, so that the hydrophobicity of the glass sheet was enhanced. However, when the concentration of the fluorinated monomer was more than 50 wt%, the contact angle of formation water on the surface of the glass sheets decreased slightly. The reason for the decrease was that when the concentration of the fluorinated monomer reached 50 wt%, the enrichment degree of the fluorinated side chains on the nanoparticles had reached a maximum. Further increasing the concentration of the fluorinated monomer would affect the stretchability of the side chains and reduce the wetting effect.

Through the above experiments, we know that the best mass ratio for the fluorinated monomer was 50 wt%. Therefore, the nano-emulsion used in the following experiments was prepared with 50 wt% tridecafluorooctyl methacrylates (G06B).

### 3.2. Characterization of Structure and Properties of Fluorinated Nanoparticles

**Figure 4:**
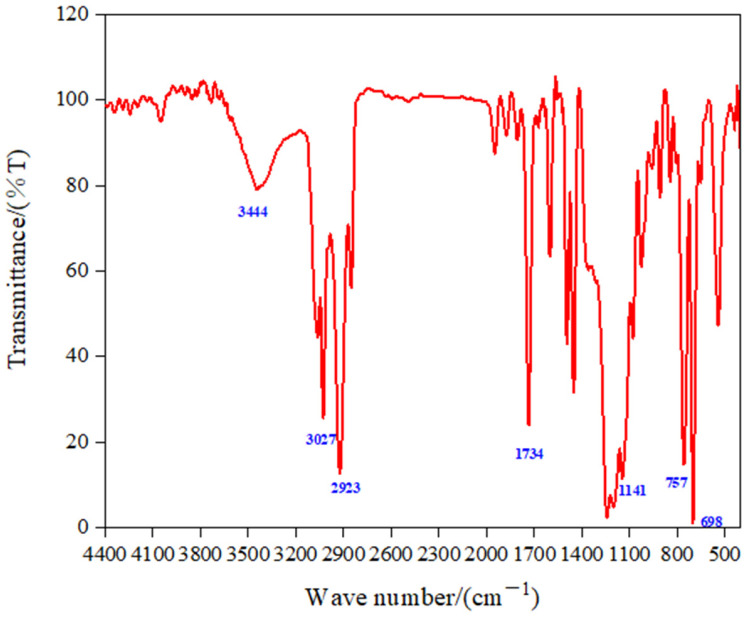
The infrared spectrum of the fluorinated nanoparticles.

The infrared spectrum of the fluorinated nano-emulsion is shown in Figure 4. The two sharp absorption peaks at 698 cm^−1^ and 757 cm^−1^ represent the C−H bond on the aromatic ring; the C−F bond stretching vibration peak appeared at 1141 cm^−1^, which proved that the fluorinated monomer G06B participated in the reaction. Affected by the fluorine atoms, the C−O bond absorption peak in the ester group had a chemical shift at 1734 cm^−1^. The stretching vibration peaks of the C−H bond were at 2924 cm^−1^ and 3027 cm^−1^; the absorption peak at 3444 cm^−1^ was the amide group. In addition, there were no out-of-plane bending vibration peaks (995 cm^−1^~985 cm^−1^ and 895 cm^−1^~885 cm^−1^) of alkene hydrogen in the spectrum, indicating that all of the monomers, crosslinkers, and initiators participated in the reaction to synthesize of the fluorinated nano-emulsion.

**Figure 5:**
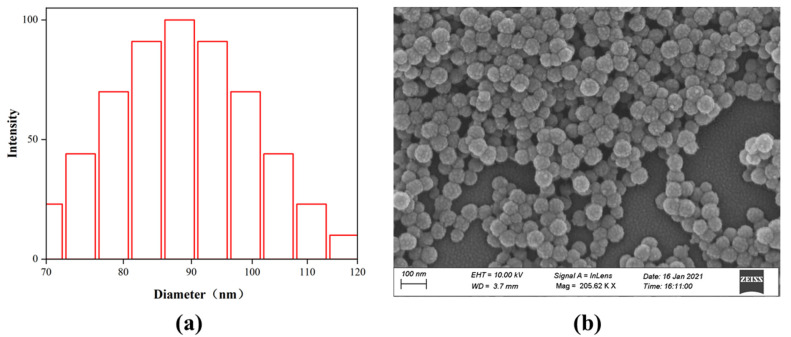
(**a**) The particle size distribution of the fluorinated nanoparticles. (**b**) The surface morphology of the fluorinated nanoparticles.

The particle size distribution of the fluorinated nanoparticles tested using dynamic light scattering (DLS) is shown in Figure 5a. The average particle size of the synthesized nanoparticles was 89.3 nm, the particle size distribution intensity at 90 nm was the largest, and most of the particle sizes were between 80 nm and 100 nm, which showed that the distribution range of the nanoparticles was quite small. The intensity ratio of the maximum particle size to the average particle size was close to 1, also indicating that the synthesized nanoparticles had a homogeneous particle size. The surface morphology of the fluorinated nanoparticles was characterized using SEM, as shown in Figure 5b; the nanoparticles were spherical particles and the surface of the particles was a papillary shape, which could make the core surface rougher.

### 3.3. Effect of Nanoparticles on the Wettability of Natural Tight Sand Cores

**Figure 6:**
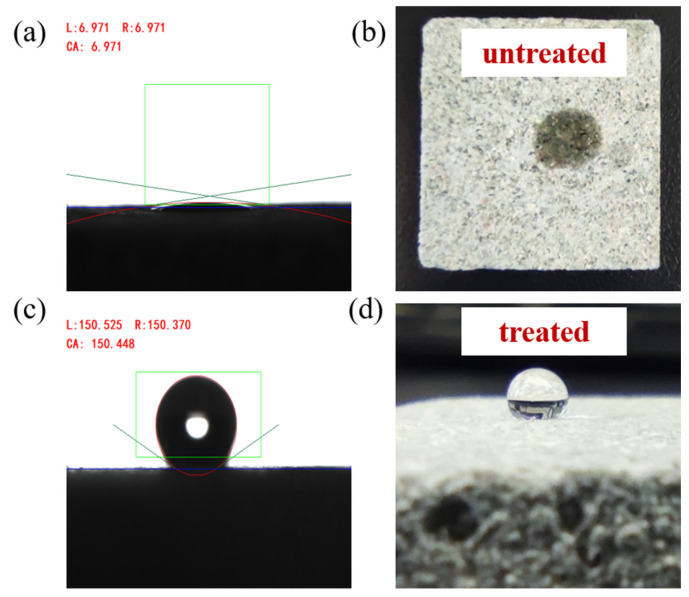
Comparison of wettability of natural cores before and after the nano-emulsion treatment: (**a**) contact angle measurement on the untreated core sheet, (**b**) outlook of a water droplet on the untreated core sheet, (**c**) contact angle measurement on the treated core sheet, (**d**) outlook of a water droplet on the treated core sheet.

The fluorinated nano-emulsion was used to alter the wettability of a natural tight sandstone core sample with a permeability of 0.02 mD, and we compared its contact angle for the formation water with that of a core sheet without nano-emulsion treatment. The results are shown in Figure 6.

The water contact angle of the untreated core sheet was 6.971°, showing that the natural tight sandstone from the Hechuan gas field was super hydrophilic. After the core was treated with the fluorinated nano-emulsion, the contact angle increased to 150.448°, which was greater than the contact angle on the glass sheet. This was mainly because the roughness of the core surface was higher than that of the glass sheet. The wettability of the core surface was obviously reversed from a hydrophilic state to a superhydrophobic state, which indicates that the nanoparticles were adsorbed on the core surface.

**Figure 7:**
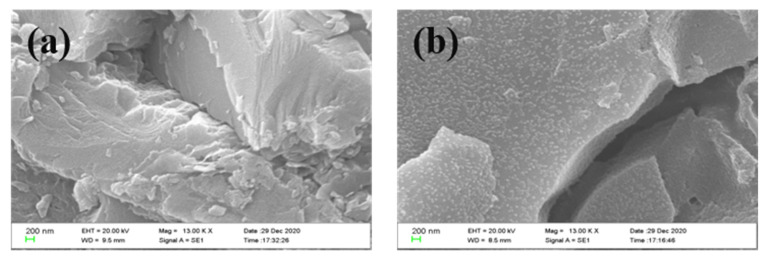
(**a**) The surface morphologies of the core before the nano-emulsion treatment. (**b**) The surface morphology of the core after the nano-emulsion treatment.

To verify the above inference, the surface morphologies of the core before and after being treated with the fluorinated nano-emulsion were characterized using SEM, which is shown in Figure 7. The surface of the untreated rock core (Figure 7a) was relatively smooth. After being treated with the nano-emulsion, a large number of fluorinated nanoparticles were attached to the surface of the core sample homogeneously (Figure 7b), which proved the interaction between the core surface and nanoparticles. Because of the existence of nanoparticles, the morphology of the core surface became rougher than the untreated one. This is part of the reason that nanoparticles can change the wettability of rocks.

**Figure 8:**
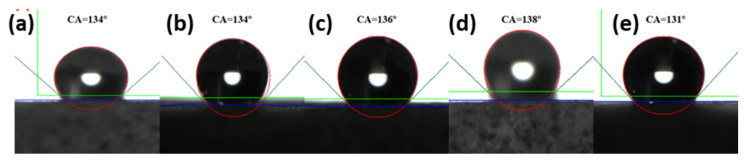
The contact angle of the treated core sheet after ultrasonic cleaning. (**a**–**e**) the contact angles on the treated core sheet after being cleaned with pure water for 5 min, 10 min, 20 min, 30 min, and 45 min.

As the fluid in the reservoir was in a high-speed flow state, the adsorption stability of the nanoparticles would determine its effect on in-field applications. Thus, we tested the adsorption stability of nanoparticles on the core surface through ultrasonic cleaning. The same core sheet from the above experiment being treated with the fluorinated nano-emulsion was ultrasonically cleaned with pure water for 5 min, 10 min, 20 min, 30 min, and 45 min, and then dried in an oven at 80 °C, and the contact angle after each cleaning and drying was measured again; the test results are shown in Figure 8a–e. The contact angles decreased, but could be maintained above 130° after ultrasonic cleaning. This was because ultrasonic cleaning removed part of the nanoparticles adsorbed on the core surface. Therefore, the contact angle was reduced, but the score sheet still had a highly hydrophobic effect.

### 3.4. Spontaneous Imbibition of Natural Tight Sand Cores

**Figure 9:**
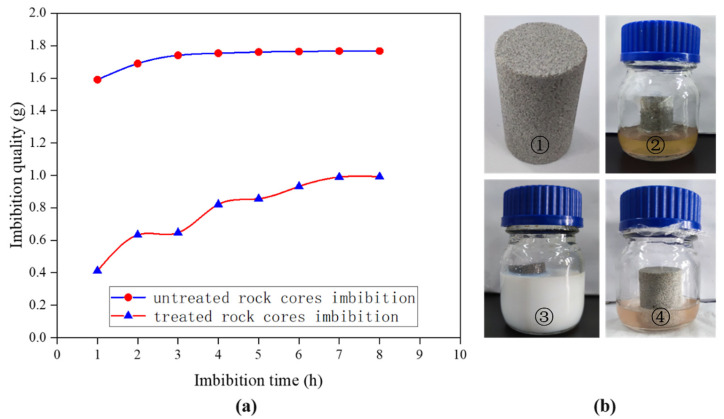
(**a**) Comparison of the spontaneous imbibition water quality of the core before and after wettability alteration. (**b**) Spontaneous imbibition experiment. ① The dry core before wettability modification. ② Spontaneous imbibition experiment of the tight sand core before wettability modification. ③ The same core soaked in the superhydrophobic fluorinated nanofluids. ④ Spontaneous imbibition experiment of the same tight sand core after wettability modification.

First, a natural and untreated core sample was used to carry out the formation water spontaneous imbibition experiment. After being dried, the same core was soaked in the fluorinated nano-emulsion to allow the nanoparticles to be adsorbed on the core surface and inside the micropores. Then, the spontaneous imbibition experiment was carried out again after being dried to compare the relationship between the water absorption quality of the core with time before and after the wettability modification. The results are shown in Figure 9.

Before and after the wettability alteration of the core, the spontaneous imbibition water quality of the core increased with time, but the water absorption quality of the tight sandstone core before treatment was significantly higher than that of the core after the wettability modification. The water absorption quality reached 1.75 g after 4 h of spontaneous imbibition for the untreated core. Further extending the experiment time, the water absorption quality almost no longer increased, indicating that the core reached spontaneous imbibition water saturation. After the same core was soaked in the fluorinated nano-emulsion and dried, the water absorption quality reached a saturation value of 0.98 g after 7 h hours of spontaneous imbibition. The water absorption quality of the core decreased by 49.7% compared with that of the core before the wettability alteration, and the water absorption rate was also significantly reduced. All of the differences were mainly due to the change in the wettability of the core from a strong hydrophilicity to superhydrophobicity; thus, the contact angle of the formation water on the core increased, which is shown in Figure 6. So, the capillary force decreased. This experiment demonstrated that the fluorinated nano-emulsion can be used to prevent water-blocking damage and delay the occurrence of the water-blocking effect.

### 3.5. Hydrophobic Mechanism of the Nano-Emulsion

**Figure 10:**
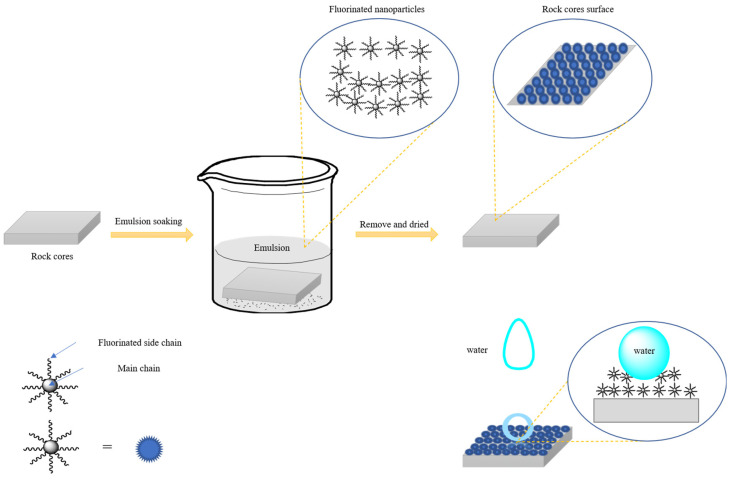
The schematic diagram of the hydrophobic mechanism of the nano-emulsion on the rock core.

The performances of the superhydrophobic nano-emulsion presented in this paper were similar to that of superhydrophobic nano-silica and other nanoparticles, such as nano-sized particle size and contact angle, but compared with nano-silica, the nano-emulsion had better a dispersion performance. By introducing a hydrophobic monomer and fluorinated monomer onto a cationic monomer, the nano-emulsion contained both hydrophobic functional groups and hydrophilic functional groups, exhibiting two affinities, which could improve its dispersing ability and stability. When the core was soaked in the nano-emulsion, the nanoparticles in the emulsion were adsorbed on the core surface and the microchannels. The adsorption forces mainly included the electrostatic force and the hydrogen bonding force. Firstly, nanoparticles can be adsorbed on the sand core through the electrostatic force between the cationic monomers and microporous channels of the sand core. In addition, the sand core contains a large number of hydroxyl groups and some broken residual bonds. When the nanoparticles were close to the core, hydrogen bonds could be formed. After the nanoparticles were adsorbed on the core, they could form the micro nanostructures on the surface of the core and its microporous channels, which increased the roughness of the core. On the other hand, the surface free energy of the core could be reduced by the fluorinated side chains. Thus, the wettability of the core could be altered. The schematic diagram of the hydrophobic mechanism is shown in Figure 10.

## 4. Conclusions

(1) A fluorinated nano-emulsion with a wettability alteration function was prepared through the soap-free emulsion polymerization method using cationic functional monomers, fluorinated monomers, and hydrophobic monomers as the main raw materials.
(2) The effect of the fluorinated monomer concentration on the hydrophobicity of the nano-emulsion was evaluated by measuring the contact angles on glass slides. Within a certain concentration range, the hydrophobicity of the nano-emulsion increased with the increase in fluorinated monomer concentration, and the optimum mass ratio of the fluorinated monomer was 50 wt%.
(3) The nano-emulsion’s wettability modification effect on the natural tight sandstone core was studied through the contact angle and spontaneous imbibition experiments. The contact angle increased from 7° to 150°, and the water absorption quality decreased from 1.75 g to 0.98 g, indicating that the wettability of the core changed from hydrophilic to superhydrophobic.
